# History of Breast Cancer in Patients with Oral Lichen Planus: A Case–Control Study

**DOI:** 10.3390/jcm13237208

**Published:** 2024-11-27

**Authors:** María García-Pola, Lucía Rodríguez-Fonseca, Claudia Llorente-Álvarez, Santiago Llorente-Pendás

**Affiliations:** 1 Department of Surgery and Medical-Surgical Specialties, Faculty of Medicine and Sciences of the Health, Oviedo University, 33006 Oviedo, Spain; luciarguezfonseca@gmail.com; 2 Hospital Rio Hortega, 47012 Valladolid, Spain; 3 Private Practice, 33004 Oviedo, Spain; llorentesantiago@gmail.com

**Keywords:** lichen planus, comorbidity, neoplasm, breast cancer

## Abstract

**Objectives**: The purpose of this study was to determine the association between oral lichen planus (OLP) and the history of cancer outside of oral cavity and the predominance of its location. **Methods**: This case–control study included 600 OLP patients and 600 control subjects evaluated in the same section, matched for age and sex to the OLP patients. OLP patients were diagnosed clinically and histologically. Initially, the prevalence of the most frequent types of cancers was described. A Pearson chi-squared test model was used to determine the association of cancer history and OLP. It was considered statistically significant whether *p* value was ≤0.05. The final multivariate regression model was built after applying a backward selection method to the complete multivariate model considering the odds ratio (OR) with a 95% confidence interval (CI). **Results**: The history of cancer was significantly associated with OLP regardless of age, sex, tobacco and or alcohol use in both univariate [OR = 2.26 (95%CI: 1.26–4.24); *p* = 0.008] and multivariate analyses [OR = 2.21 (95%CI: 1.21–4.19); *p* = 0.012]. According to the location of cancer, there was an association between OLP and history of breast cancer [OR = 3.71 (95%CI = 1.03–13.38); *p* = 0.032]. **Conclusions**: This case–control study suggests a higher frequency of cancer, particularly breast cancer, among patients with OLP compared to the control group. Due to the study’s design and sample limitations, these findings should be interpreted cautiously. Future longitudinal, multi-institutional studies with rigorous control for cancer history and other confounding factors are essential to further explore the association between OLP and cancer, particularly breast cancer.

## 1. Introduction

Oral lichen planus (OLP) is considered as a chronic inflammatory disorder and its global prevalence is estimated at 1.01% [1]. OLP mostly occurs in middle-aged individuals and is more common in women. OLP is an oral potentially malignant disorder [2] with a pooled proportion of malignant transformation of 1.43% [3].

Despite past and recent research, the pathogenesis of OLP remains unknown, where T cells play a relevant role in managing immune response [4]. OLP is not an infectious disease, but it has been considered that some antigens that present primarily to Langerhans cells may come from the oral microbiota [5]. It has also been linked to genetic factors and to an association with the hepatitis C virus, systemic psychosomatic disorders, or to endocrine, cardiovascular and autoimmune diseases [6].

The antigens involved in the pathogenesis of OLP trigger the start of T-cell proliferation and immune response against basal epithelial cells [7]. Physiologically, these basal epithelial cells suffer from apoptosis by the disruption of their basement membrane and the lamina propria, with intra- and subepithelial T-lymphocyte infiltration [8].

In the pathogenesis of OLP, T cells intervene through pro-inflammation and the suppression of inflammation [9]. The attraction of other cells as well as the secretion of molecules such as cytokines and chemokines will favor the progression or regression of the disease [8,10,11].

Chronic inflammation at the expense of cytokines could favor cell proliferation and its progression to oral squamous cell carcinoma (OSCC) [12]. Inflammation has also been described as one possible route for the pathogenesis of other types of cancer [13]. In this process, pro-inflammatory mediators act by stimulating inflammation to either slow or stop tumor progression or to facilitate tumor growth and metastasis [14]. Furthermore, the increase in proteins, such as CXCL13, that promote inflammation and genes related to Th-17 cell differentiation have been identified in OLP patients, in OSCC [15] and in other carcinomas, such as breast [16,17], colorectal [18], bladder [19] and cutaneous melanoma [20].

When questioning the patient with OLP regarding their medical history, it is not very common to include investigations on their history of having suffered cancer outside the oral cavity. Thus, questions are collected about whether the patient has suffered from “previous malignant diseases” [21], “neoplastic diseases” [22,23], “disease of cancer” [24], “non-oral malignancy” [25] or “comorbidities of neoplastic pathologies” [26].

The prevalence of cancer history in OLP patients has been reported in some research, providing percentages in a general way that refer to “any type of cancer” in the global population, or specifying by pointing out the exact location outside the oral cavity. Thus, it has been pointed out that the rate of oropharyngeal cancers can rise from 0 [27] up to 22.5% in oral locations [28]. Variations in the prevalence of cancer history within the same country have also been expressed, such as in Italy, from 2.4% [23] to 21% [29], or in Sweden, from 0.4% [30] to 4.76% [31].

The types of cancer in patients with OLP include colon (2.6%) [28], skin (3.8%) [28], head–neck–face (3.8%) [28], hematological (1.9%) [28], hepatic (10.1%) [32] or thyroid, from 1.2% [28] to 5.8% [33]. In OLP patients, it has also been noted that prostate cancer represents 0.7% [31] or 0.8% out of all malignancies [28]; however, among men, it is between 2.27% [31] and 2.63% [28]. Breast cancer prevalence varies from 0.9% [34] to 7.1% [18] among all cancers, and among women, it can represent up to 9.3% [28].

Methodologically, the research mentioned in the previous paragraph belongs to studies with a small sample size, cohort [22,24,29] or case–control studies [21,26,28]. In other studies, even though they contain large samples of OLP, they are not compared with control groups [23,31].

Because of this variability about the registration of the prevalence of the history of cancer in OLP and the possibility of history of cancer to act as a risk of suffering from lichen planus, we consider the need to carry out a case–control study. Therefore, the purpose of the present study was to survey the link between OLP patients and a history of cancer through a case–control study, to ascertain the relationship between history of cancer and OLP with the following research question: “Is a history of cancer outside of the oral cavity associated with the risk of suffering from Oral Lichen Planus?”.

## 2. Materials and Methods

The methodological design was a retrospective case–control study planned according to the Strengthening Reporting of Observational Studies in Epidemiology (STROBE) guidelines (Table S1) [35].

### 2.1. Subjects

The experimental group consisted of 600 OLP patients cared for in the Oral Medicine Section at Oviedo University, between January 2014 and December 2023. The study was approved by the Ethics Committee of the Principality of Asturias and complied with the requirements of the Declaration of Helsinki (nº 127/13 and nº 140/23).

The sample size was limited to a 10-year period without considering a specific number of subjects. Thus, the inclusion of patients was consecutive and without prior selection to reduce the risk of selection bias. The criteria for inclusion in the case group was for patients to be at least 18 years old and to have a diagnosis of OLP appraised clinically and histologically according to the WHO criteria [36] with subsequent modifications [37], and as described in our previous research [38]. Therefore, inclusion criteria for clinical diagnosis of OLP were contemplated according to the following criteria: lesions with gray-white lines or papular pattern and by atrophic, erosive, bullous and/or plaque variety lesions of a two-sided disposition. Histopathological criteria included the following: (1) “liquefactive degeneration” in the basal layer; (2) a band-like zone of cellular infiltration limited to the top part of the lamina propria; and (3) an absence of epithelial dysplasia [37].

**Figure 1:**
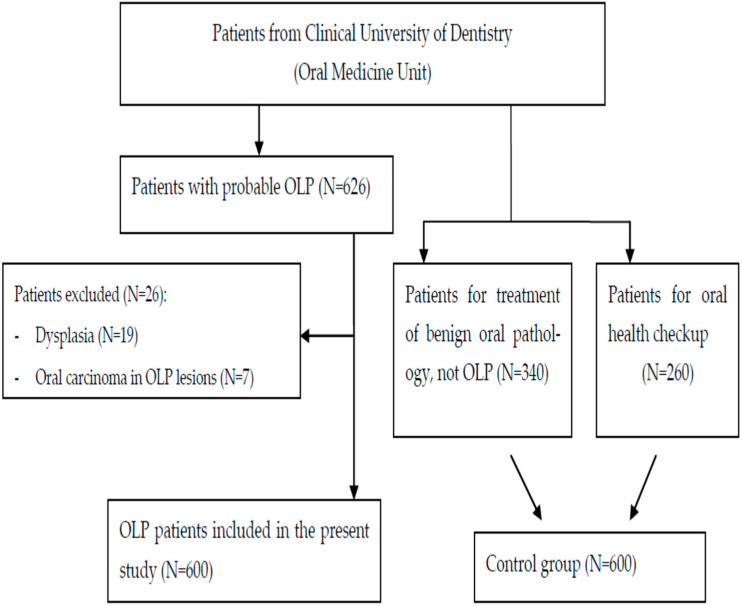
Flow chart of the selections of patients included in the present study. Oral lichen planus (OLP).

The control group consisted of 600 consecutive patients matched for age and sex to the OLP patients (Figure 1). The subjects of the control group were selected consecutively until completing the same number of patients with the same age and sex corresponding to the OLP group. They were evaluated in the same section over the same interval of time for other types of benign oral mucous diseases, without any signs or previous history of OLP (N = 340), or for examination and checkup of the oral cavity (N = 260) (Figure 1). We considered benign oral mucous diseases, traumatic lesions, oral non-pathological lesions and lesions treated with excisional biopsy such as mucoceles, diapneusia, epulis *fissuratum*, fibroepithelial hyperplasia or other connective tissue, squamous papilloma and lipomas. Oral non-pathological lesions were included following the criteria of Tortorici et al.: Fordyce granules; coated tongue; and hairy tongue [39].

The exclusion criteria of the case group were subjects under 18 years old, either pregnant or breastfeeding, presenting oral lichenoid reaction or oral cancer in addition to the OLP on the day of the first visit, or patients who had undergone radiotherapy or taken anti-cancer drugs over the previous months. Diagnosis lichenoid reaction was considered based on the criteria by Aguirre et al. [40]. Patients with OLP outside the oral cavity were not excluded.

### 2.2. Protocol

The protocol included recording patients’ sex, age, and tobacco and alcohol consumption. The smoker variable was divided into 5 categories: non-smoker, with never having smoked; smoking habit with ≤10 cigarettes; smoking habit with more than 10 cigarettes [30,41]; other forms of smoking (such as cigar, pipe or another); and former smoker, where the patients had stopped smoking at least 6 months before interview [42]. We considered unit of alcohol as follows: 1 glass of wine, ¼ liter of beer, or another measure of liqueur [41,43]. Therefore, the drinker variable was divided into 4 categories: non-drinker (never); consumption of up to two units per day; consumption of more than two units; and weekend drinker [41,43,44].

Clinical findings included the form of OLP (atrophic-erosive and non-atrophic-erosive), number of locations, medical history, and prescription medication. The number of oral locations recorded was two, consistent with the bilateral definition [36,37], or more [38]. Before performing the incisional biopsy on the first visit, all patients were interrogated about their history of cancer treatment or prior diagnoses of cancer, providing where necessary the document of their clinical report.

Data collection was always carried out by the first author, accompanied at certain times by the co-authors, following the usual protocol for clinical history, anamnesis, and examination. 

### 2.3. Statistical Analysis

Statistical analysis was performed using R-core (R Development Core Team, version 4.1.3). Initially, a descriptive study was made of each variable. Due to the mean age of the patients registered, the dichotomous cut-off point was split into two categories: 60 years old or under, and over 60. Tobacco and alcohol consumption were divided, respectively, into the 5 and 4 categories considered previously. The clinical form was distributed based on non-atrophic-erosive and atrophic-erosive following the criteria of Carbone et al. [23] and our previous study [38]. The number of OLP locations was divided into two locations (bilateral by definition) [37], and three or more locations [38]. Pearson’s Chi^2^ test and Fisher test were used to compare categorical parameters between the groups. *p* values below 0.05 were considered statistically significant. Logistic regression models were used to measure the association of OLP and history of cancer. Given the different types of cancer according to sex, and that age > 60 years, tobacco use, and alcohol intake are considered risk factors for several cancers, these variables were considered in the multivariate analysis [45] to estimate the odds ratios (ORs) and their 95% confidence intervals (95% CIs). The final multivariate regression model was built after applying a backward selection method to the complete multivariate model. The Akaike information criterion (AIC) was used to measure the quality of the stepwise regression model.

## 3. Results

### 3.1. Descriptive Analysis

#### 3.1.1. Age and Sex

From a sample of 1200, the OLP patients and the control group each consisted of 443 women (73.8%) and 157 men (26.2%) (Table 1). The age fluctuated from 18 to 90 years old, with a mean age of 59.58 (±12.47). At the time of the first visit, the mean age of the women was 60.59 (range 18–90) and the mean age of the men was 56.71 (range 18–86). 

#### 3.1.2. Tobacco Use and Alcohol Drinker

There were fewer smokers and more ex-smokers in the OLP group than in the control group (11.2% vs. 24% and 14.8% vs. 13.5%, respectively).

Regarding alcohol consumption, when comparing the two groups, there were more patients among the OLP group who drank more than 2 units per day (8.9% vs. 7.2%), and the control group had a higher number of patients who were weekend drinkers (10.4% vs. 4%).

#### 3.1.3. Clinical Number of Oral Locations of OLP and Clinical Form of OLP

Three hundred and twelve OLP patients (52.0%) had two locations of OLP and three hundred and sixty-eight (61.3%) of OLP patients presented the atrophic-erosive clinical form (Table 1).

#### 3.1.4. Prevalence of History of Cancer and Types of Cancer

A total of 35 OLP patients had a history of cancer (5.8%), compared to 16 in the control group (2.7%). The demographic characteristics of patients with a history of cancer in the OLP and the control group are shown in Table 2. Forty-two of them were women, twenty-nine (69.07%) and thirteen (81.25%) for each group, respectively. The mean age of the OLP patients with a history of cancer was 62.13 (35 to 81), whereas in the control group, it was 61 (43 to 75). 

The clinical form of OLP with history of cancer was atrophic-erosive in 25 patients (71.42%) and 19 patients presented 3 or more locations (54.3%).

OLP patients had been diagnosed with cancers in fourteen locations, with the most common cancers in the OLP patients being breast cancer (1.83%) and cancer of the cervix (1.16%). In the control group, the two most common cancers were breast and basal cell carcinoma (0.5%). One patient in each group reported a history of cancer in two locations. The types of cancer and demographic characteristics according to age and sex are reflected in Table 3.

### 3.2. Results of the Univariate and Multivariate Analyses

According to the type of cancer, there were statistically significant differences between the OLP patients’ group and the control group for breast cancer with OR = 3.71, CI = 1.03–13.38; *p* = 0.032. Despite the higher prevalence of cervix cancer and colon cancer among the OLP patients, they did not show statistically significant differences (*p* = 0.094 and *p* = 0.317, respectively). 

The results of the univariate and multivariate analyses of history of cancer are presented in Table 4. The crude OR associated was 2.26 [95% confidence interval (CI) 1.26–4.24; *p* =0.008], and the multivariate after applying a backward selection showed an association (OR = 2.21, CI = 1.21–4.19; *p* = 0.012). Age, gender, smoking and alcoholic habits are not predictive variables in the relationship between cancer history and OLP patients. The difference in the values recorded when using AIC was minimal in the different steps when introducing the variables of sex, age, tobacco and alcohol (Table 5).

## 4. Discussion

The results of the present observational study suggest a link between OLP and a history of cancer outside the oral cavity. Our case–control study showed that OLP patients were twice as likely to have a history of cancer than subjects without OLP (OR: 2.21). We have described a heterogeneous sample in the types of cancer as did Dave et al. [28] and Rödström et al. [31].

The sample of OLP patients follows the most frequently recorded characteristics of OLP, i.e., a higher frequency among women [46,47,48,49] and an age approaching 60 years, as recorded in previous studies [50,51,52].

Regardless of the number of cigarettes per day, there were more smokers in the control group when compared to OLP patients in a statistically significant way. These findings corroborate previous studies [21,53,54] and contradict others [26,55,56].

In our sample of patients with OLP, there were fewer subjects who drank in a statistically significant manner. Pippi et al. stated that when the variable of smoker and drinker was considered together, it was higher in patients with OLP than in the control group [57]. Other studies have shown that there are no differences between the alcohol habits of patients with OLP and the control group [32,54,56].

The main objective of this research was to provide the prevalence of cancer history in patients with OLP, showing a prevalence of 5.8%, which contrasts with the control group of 2.7%. It is understood that this epidemiological study cannot prove that cancer history and OLP are causally related, nor that OLP patients are more susceptible to presenting cancer outside the oral cavity, but it does clarify their association. 

Two decades ago, Rödström et al. described a prevalence of 4.76% of history of cancer in a sample of 1080 Swedish patients with OLP, from the city of Göteborg; however, these data were lower than those expected on the incidence of cancer in the general population [31]. In the same city, in 2011, a lower prevalence of cancer history, of 0.4%, was observed among OLP patients, while in the general population, it was 3.4% [30]. These figures contrast with those provided by Burkhart et al. [58] on a sample of North Americans in 1996, who recorded 16%, and more recently, in 2023, the prevalence reached up to 22.5% [28], thus observing an increase in prevalence with data subtracted from a database.

Other figures on OLP prevalence were published in a comparative study on the characteristics of OLP in Croatian and Australian patients, with history of cancer recorded at 3.7% in the first and 17.8% in the latter country [59], which shows a geographical discrepancy. Another example is from Israel, where a history of cancer was recorded in 14.9% of a sample of 171 OLPs [25].

The prevalence of history of cancer in OLP patients also varies widely within the same country; for example, in Turin, Italy, a rate of 2.47% was registered [23]; in Brescia, 5.6% [22] (a percentage very similar to that recorded in the present study (5.8%)); in Catanzaro, it was 7.9% [26]; and in Rome, it was 21% [29]. Geographically, the highest prevalence of a history of cancer in patients with OLP has been recorded in the United States [28,58], Brisbane (Australia) [59] and the aforementioned Italian city of Rome [29]. 

Certain factors are recognized that may affect the incidence of cancer, highlighting that the type of cancer is dependent on age > 60 years, sex, tobacco and alcohol [45]. By considering the sex of the patients, the present study confirmed the figures on the prevalence of cancer proposed by the research of a cohort of 808 patients from Northern Italy [23]. In both studies, the absence of prostate cancer was found among men with OLP, while in both genders, the prevalence of breast cancer among women with OLP was exactly 2.4% (12 breast cancer among 493 Italian women and 11 among Spanish women). In addition, our study contributed an OR of 3.7 when compared with patients without OLP. These figures contrast with the reported percentage of breast cancer patients with OLP of 9.3% in North America, the high-income country with the highest incidence of breast cancer [60].

On the contrary, we did not find an association of cancer history in OLP patients with risk factors associated with sex, age over 60 years, smokers and drinkers. These findings may be in agreement with the reflection that patients over 60 years of age do not have a higher incidence of cancer than in young people based on the mentioned factors [45].

Other factors that could be involved in the association of cancer history and OLP that justify the geographical discrepancies are related to genetic factors, lifestyle, and comorbid diseases such as viral, or immunological diseases [45,61]. Upon analyzing the types of cancers associated with OLP, there are genetic and non-genetic factors for breast cancer [62]; among the latter, excess body weight stands out [63]. Adamo et al. showed that body mass index was higher in OLP patients than in the control group in a statistically significant way [64]; however, in a later study, they observed that keratotic forms of OLP did not present this association [21]. 

Regarding viral diseases, the risk of a history of liver disease in OLP patients may also be due to geographical and pathogenic factors through its link with hepatitis C virus (HCV) [65]. We presented one case of a history of hepatocellular carcinoma in an OLP patient, with a figure much lower than those presented in a Japanese population [32]. The study by Nagao et al. [32] took place in Japan, and the authors highlighted that the study was carried out in a city whose residents had a high prevalence of HCV infection. These authors reported 8 hepatic carcinomas in a sample of 59 patients (13.5%), while in the control group of 85 subjects, they observed 2 cases of hepatic cancer (2.35%) [32]. On the contrary, they showed that other extrahepatic carcinomas were more frequent in the control group than in the OLP group (11.76% vs. 8.47%) [32]. This HCV association with cancer would justify the involvement of more pathogenic factors related to cancer than those mentioned as major ones depending on the location. Although the main cause of hepatocellular carcinoma in some countries such as Japan and the United States is HCV [66], recent studies have indicated that the incidence of hepatitis C-related liver cancer is decreasing [67,68]. 

Another carcinoma related to OLP was thyroid cancer, being geographically more evident in prevalence in Taiwan (1.88%) [33], the United States (1.28%) [28] and Spain (0.33%). Comorbidity with thyroid pathology has been observed in patients with OLP, reflecting its autoimmune basis [69], non-immune inflammatory processes [70], and raising the question of whether the comorbidity could be due to genetic susceptibility. Thyroid cancer is also associated with obesity and dyslipidemia and other cardiovascular risk factors [71]. However, a recent systematic review has shown that patients with OLP have higher levels of total cholesterol and lower high-density lipoprotein than the control group, but no other parameters in particular, which makes it difficult to interpret the link between thyroid cancer, dyslipidemia and OLP [72].

A type of tumor present in our sample was thymoma. The recent literature indicates the possible association between thymoma in isolation [73,74] or as part of Good’s syndrome [75], drawing attention to the thought of a thymoma in the face of erosive OLP.

The possible link between a history of cancer and LP is complicated to explain. In OLP, there is an immune response of Th-1, Th-9 and Th-17 lymphocytes with an imbalance of Th-2 and subsequently an increased cytokine release [7,76]. Cytokines produced by inflammatory Th-17 cells stimulate inflammation-dependent tumor growth by promoting angiogenesis and suppressing antitumor immunity [77], a condition that has been described in skin [78], breast [79,80] and papillary thyroid carcinoma tumors [81]. However, recently, Chen et al. observed a negative evaluation between the expression of IL-17 levels during the pathogenesis of OLP and determined an aberrant expression of fibroblast growth factor 21 (FGF21), a protein also related to autoimmune diseases (ADs) [82]. Other studies have indicated that some ADs such as Sjogren’s syndrome or lupus erythematous may act as protective factors against breast cancer risk [83].

In a systematic review that analyzed the malignancy of OLP, it was revealed that the history of cancer could not be adequately included due to a lack of information in the studies selected for analysis [84]. The present study is the starting point to emphasize the variable history of cancer among patients with OLP. Considering the fact that OLP is a potentially malignant disorder, clinicians will benefit from being aware of this link, which also suggests the benefits of oral screening for OLP in women with breast cancer.

The present study has some limitations. Methodologically, the main limitation of this case–control study is related to the fact that the control group belongs to subjects from the same unit as patients with OLP, even though we selected a control group matched by sex and age and carried out our study with a large sample. In this sense, the methodological criticism of case–control studies developed by Seyghan et al. [85] is proven, as the authors argue that not even the control group from the general population can be valid, since people from the general population could possibly have a lower prevalence of the pathology under study—in this case, cancer. In addition, to mitigate confounding factors, a multivariate logistic regression was performed [86,87].

Despite including the most representative risk factors for oral cancer, there are others such as economic income, healthcare or even diet, which could be included in future studies.

The study was conducted in a single center and using controls from the same section without OLP, so we suggest new multicenter studies with general population controls to reduce prevalence biases as well as prospective cohort studies that collaborate in the deepening of this topic. Another limitation is that the ranges of the confidence intervals recorded for the association between cancer history and particularly breast cancer are close to the null value, so they do not show a solid robustness even though the association findings are statistically significant. Therefore, these results should be interpreted with caution, suggesting new studies, ideally prospective, to confirm the possible association of OPL and cancer history.

## 5. Conclusions

This case–control study suggests a higher frequency of cancer, particularly breast cancer, among patients with OLP compared to the control group. Due to the study’s design and sample limitations, these findings should be interpreted cautiously. Future longitudinal, multi-institutional studies with rigorous control for cancer history and other confounding factors are essential to further explore the association between OLP and cancer, particularly breast cancer.

## Figures and Tables

**Table 1:** Demographic characteristics of oral lichen planus (OLP) patients and control group; C: cigarette; U: units; *: statistically significant (*p* < 0.05). ^#^: Pearson’s Chi^2^ test.

Variable		OLP (%)	Control (%)	*p* Value ^#^
Gender	Male	157 (26.2)	157 (26.2)	1
	Female	443 (73.8)	443 (73.8)	
Age	Mean (SD)	59.58 (±12.47)	59.58 (±12.47)	1
Tobacco use	No	444 (74.0)	375 (62.5)	
	Categories			
	≤10 c	30 (5.0)	67 (11.2)	<0.001 *
	>10 c	36 (6.0)	75 (12.5)	<0.001 *
	Cigar	1 (0.2)	2 (0.3)	0.482
	Former smoker	89 (14.8)	81 (13.5)	0.658
Alcohol drinker	No	476 (79.3)	445 (74.2)	
	Yes	124 (20.7)	155 (25.8)	0.034 *
	≤2 u	47 (7.8)	49 (8.2)	0.611
	>2 u	53 (8.9)	43 (7.2)	0.511
	Weekend	24 (4.0)	63 (10.4)	<0.001 *
Atrophic-erosive form	No	232 (38.7)		
	Yes	368 (61.3)		
Oral location	2	288 (48.0)		
	≥3	312 (52.0)		
History of cancer	No	565 (94.2)	584 (97.3)	
	Yes	35 (5.8)	16 (2.7)	0.008 *

**Table 2:** Demographic characteristics of oral lichen planus (OLP) patients and control group; C: cigarette; U: units. ^#^: Pearson’s Chi^2^ test; ^†^: Test de Fisher.

Variable		OLP (%)	Control (%)	*p* Value
Gender	Male	6 (66.67)	3 (33.33)	1 ^†^
	Female	29 (69.05)	13 (30.95)	
Age	≤60	12 (60.0)	8 (40.0)	0.286 ^#^
	>60	23 (74.19)	8 (25.81)	
Tobacco use	No	25 (75.76)	8 (24.24)	0.189 ^†^
	Categories			
	≤10 c	1 (50.0)	1 (50.0)	
	>10 c	1 (25.0)	3 (75.5)	
	Former smoker	8 (66.67)	4 (33.33)	
Alcohol drinker	No	29 (70.73)	12 (29.27)	0.912 ^†^
	≤2 u	2 (66.67)	1 (33.33)	
	>2 u	2 (50.0)	2 (50.0)	
	Weekend	2 (66.67)	1 (33.33)	

**Table 3:** Type of cancers in patients with OLP and the control group with history of cancer. ^1^. Patient with two types of cancer location. In square brackets, ranges of age. In parentheses, percentages.

Type of Tumor	OLP F/M	CG F/M	OLP Mean Age [Range]	CG Mean Age [Range]
Breast	11/0	3/0	66.4 [57–77]	65.3 [55–75]
Skin				
Melanoma	1/0	1/0	72	38
Basal carcinoma	3/0	1 ^1^/2	61.6 [43–81]	59.6 [43–70]
Colon	1/2	1/0	54.3 [35–66]	61
Small intestine	0/1		67	
Thyroid	2/0		52 [50–54]	
Cervix	7 ^1^/0	2/0	65.1 [53–71]	70.5 [66–75]
Ovary	2/0		73.5 [71–76]	
Lymphatic	1/0	2/0	58	62 [57–67]
Thymus	1/0		46	
Stomach	0/1	2/0	62	56.5 [55–58]
Vocal cord	0/1		67	
Hepatocellular carcinoma	1/0		63	
Peritoneum	1 ^1^/0		62	
Bladder		0/1		65
Rectum		1 ^1^/0		66
Nail		1/0		61
Total	30 (85.71)/	13 (81.25)/		
	5 (14.29)	2 (18.75)		

**Table 4:** Results of the univariate and multivariate analyses. OR: Odds ratio; CI: confidence interval (95%); SD: standard derivation; *: statistically significant (*p* < 0.05).

Variable	OLP n (%)	Control Group n (%)	OR Univariate (CI, *p* Value)	OR Multivariate (CI, *p* Value)
Sex				
Female (%)	443 (50.0)	443 (50.0)	-	-
Male (%)	157 (50.0)	157 (50.0)	1.00 (0.77–1.29, *p* = 1.000)	1.10 (0.84–1.44, *p* = 0.478)
Age				
≤60	292 (50.0)	292 (50.0)	-	-
>60	308 (50.0)	308 (50.0)	1.00 (0.80–1.25, *p* = 1.000)	1.00 (0.77–1.29, *p*= 0.884)
Tobacco use				
No (%)	444 (54.2)	375 (45.8)	-	-
≤10 c (%)	30 (36.9)	67 (69.1)	0.38 (0.24–0.59, *p* < 0.001 *)	0.42 (0.26–0.67, *p* < 0.001 *)
>10 c (%)	36 (32.4)	75 (67.6)	0.41 (0.26–0.61, *p* < 0.001 *)	0.41 (0.26–0.63, *p* < 0.001 *)
Cigar (%)	1 (33)	2 (66.7)	0.42 (0.02–4.42, *p* = 0.482)	0.91 (0.64–1.29, *p* = 0.592)
Former smoker (%)	89 (52.4)	81 (47.6)	0.93 (0.67–1.29, *p* = 0.658)	0.39 (0.02–4.07, *p* = 0.439)
Alcohol drinker				
No (%)	476 (51.7)	445 (48.3)	-	-
≤2 u (%)	47 (49.0)	49 (51.0)	0.90 (0.59–1.37, *p* = 0.611)	1.04 (0.67–1.62, *p* = 0.844)
>2 u (%)	53 (55.2)	43 (44.8)	1.15 (0.76–1.76, *p* = 0.511)	1.36 (0.87–2.13, *p* = 0.179)
Weekend (%)	24 (27.6)	63 (72.4)	0.36 (0.21–0.57, *p* < 0.001 *)	0.44 (0.26–0.73, *p* = 0.002 *)
History of Cancer				
No (%)	565 (49.2)	584 (50.8)	-	-
Yes (%)	35 (68.6)	16 (31.4)	2.26 (1.26–4.24, *p* = 0.008 *)	2.21 (1.21–4.19, *p* = 0.012 *)

**Table 5:** Logistic regression models to analyze differences for “history of cancer or not” and the following variables: alcohol drinker, tobacco use, age, sex. AIC: Akaike information criteria; df: degrees of freedom.

Model	Df	Deviance	AIC
<none>		1607.8	1625.8
History of cancer	1	1614.6	1630.6
Alcohol drinker	3	1621.7	1633.7
Tobacco use	4	1635.9	1645.9
Age	1	1607.8	1625.8
<none>		1607.7	1627.7
History of cancer (yes/no)	1	1614.6	1632.6
Alcohol drinker	3	1621.5	1635.5
Tobacco use	4	1635.9	1647.9
Sex	1	1607.7	1627.7
Age	1	16.7.7	1627.7
<none>			
History of cancer (yes/no)	1	1614.5	1634.5
Alcohol drinker	3	1621.2	1637.2
Tobacco use	4	1635.9	1649.9

## Data Availability

The data presented in this study are available upon request from the corresponding author.

## Supplementary Materials

The following supporting information can be downloaded at: https://www.mdpi.com/article/10.3390/jcm13237208/s1, Table S1: STROBE statement—checklist of items that should be included in reports of case–control studies.
